# Supplementary material for: The Anionic Polymerization of a tert-Butyl-Carboxylate-Activated Aziridine
Source: Polymers (Basel). 2022 Aug 10;14(16):3253. doi: 10.3390/polym14163253 (PMC9416489; doi:10.3390/polym14163253)
Supplement: Supplementary file 1 [file polymers-14-03253-s001.zip › polymers-1839527-supplementary.pdf]

Supporting information

The Anionic Polymerization of a *tert*-Butyl Carboxylate Activated Aziridine

Chandan Giri, Jen-Yu Chang, Pierre Canisius Mbarushimana and Paul A. Rupar *

Department of Chemistry and Biochemistry, The University of Alabama,
Tuscaloosa, AL 35487-0336, USA
* Correspondence: parupar@ua.edu

NMR spectra……………………….………………………….…………………...S2

GPC traces..………..………………………………… …..…………………………..S6

MALDI-TOF MS…….……………………………...………………..………………..S10

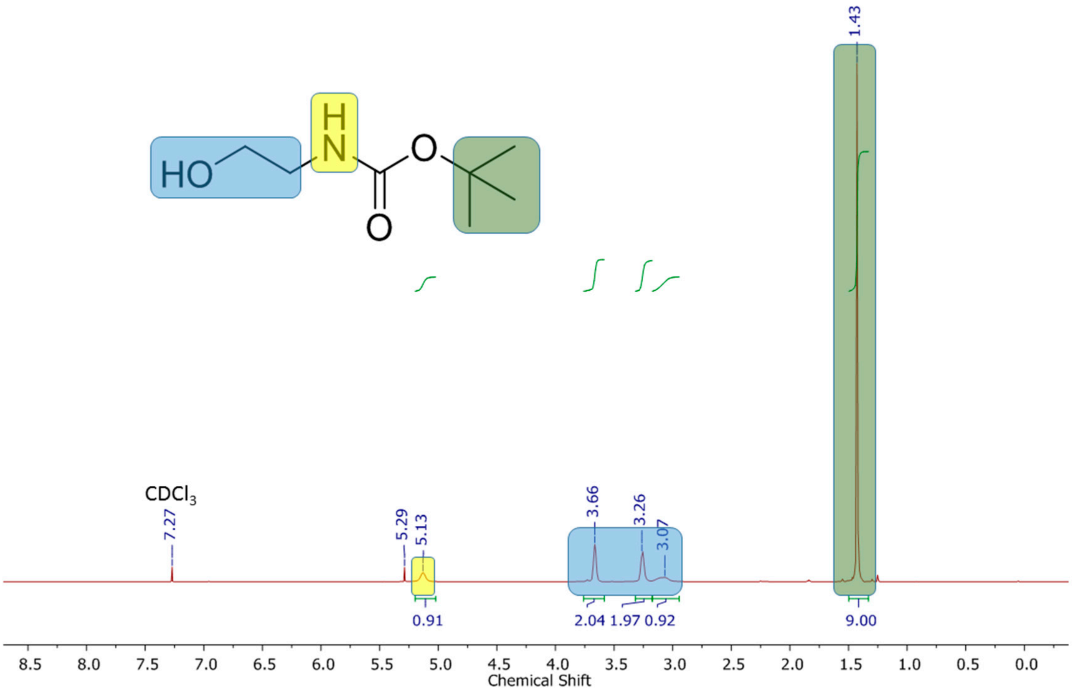

Figure S1. ^{1}H NMR spectrum ($CDCl_3$) of *tert*-butyl (2-hydroxyethyl)carbamate.

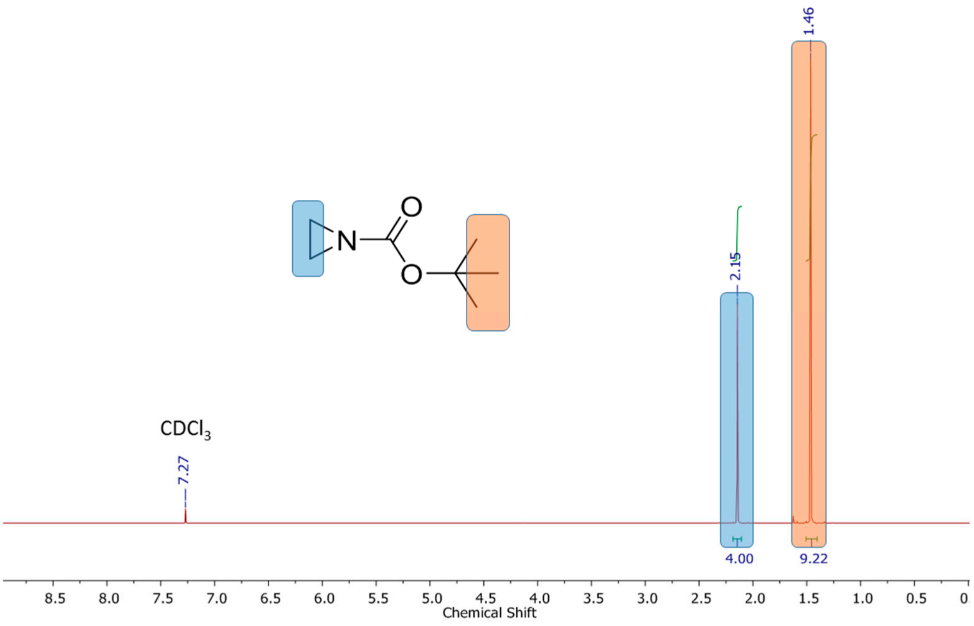

Figure S2. ^{1}H NMR spectrum ($CDCl_3$) of **BocAz**.

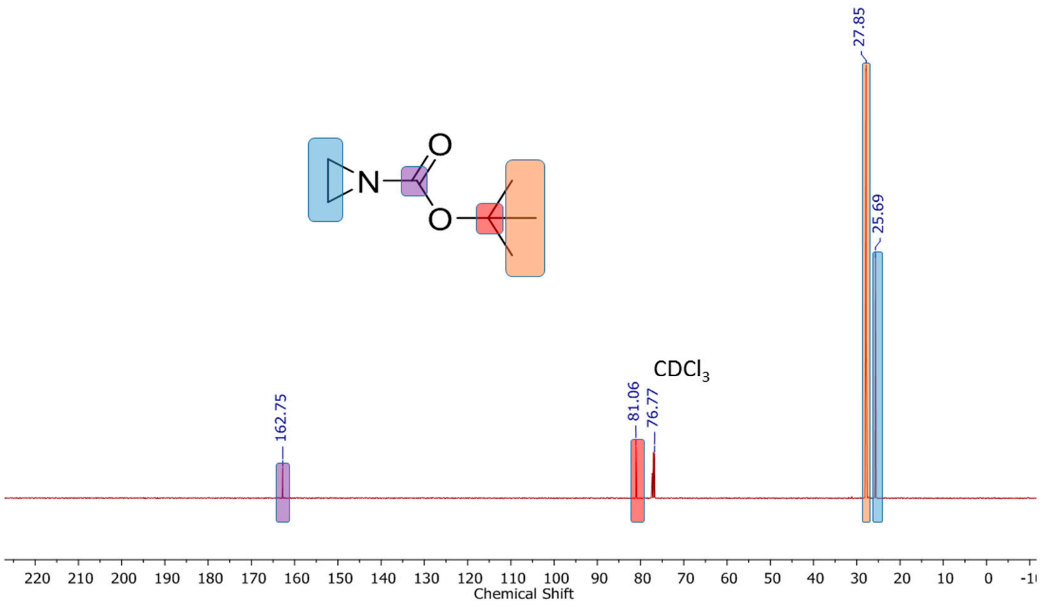

Figure S3. ^{13}C NMR ($CDCl_3$) spectrum of **BocAz**.

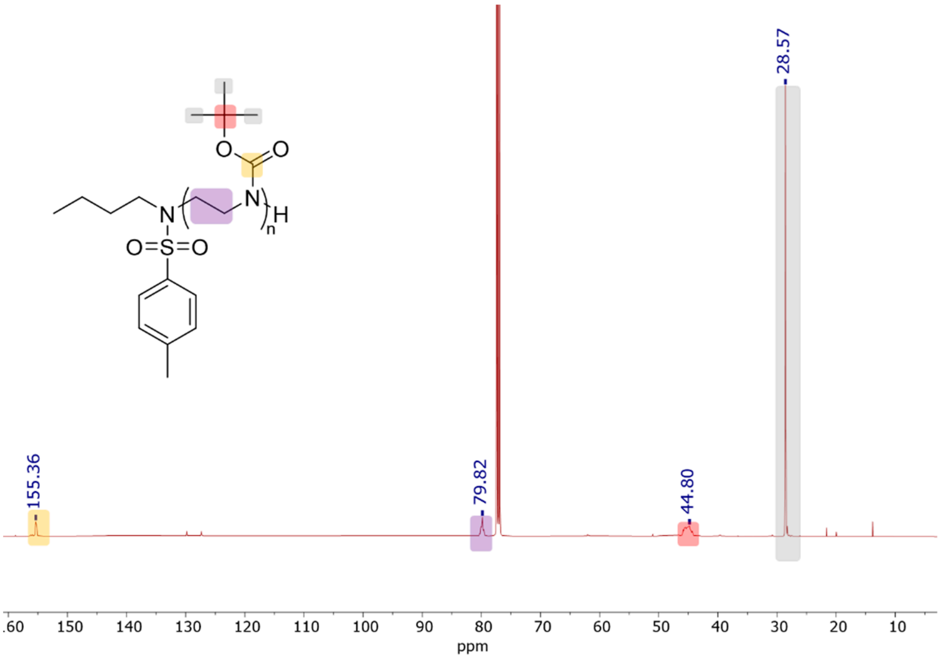

Figure S4. ^{13}C NMR spectrum ($CDCl_3$) of poly(BocAz). The unlabeled, low intensity, sharp signals between 10 and 25 ppm as well as those near 130 ppm are believed to arise from the initiator.

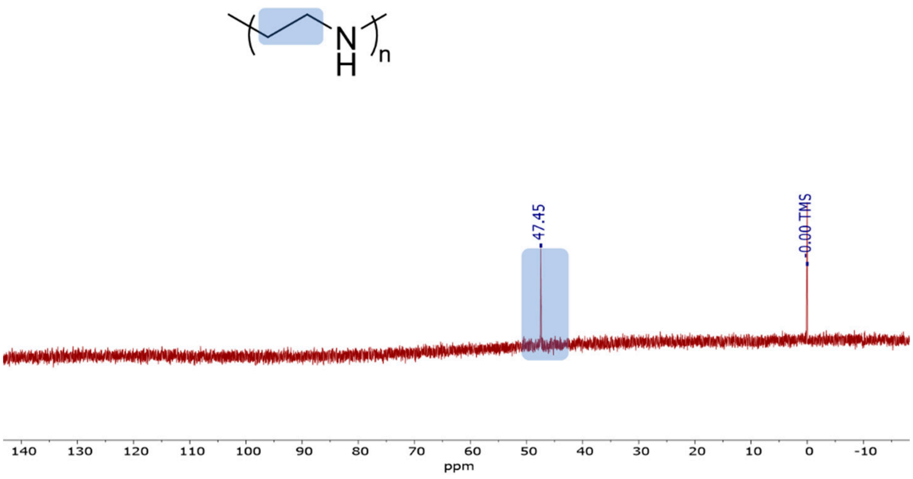

Figure S5. ^{13}C NMR (D_2O) spectrum of linear PEI from the deprotection of poly(BOCAz).

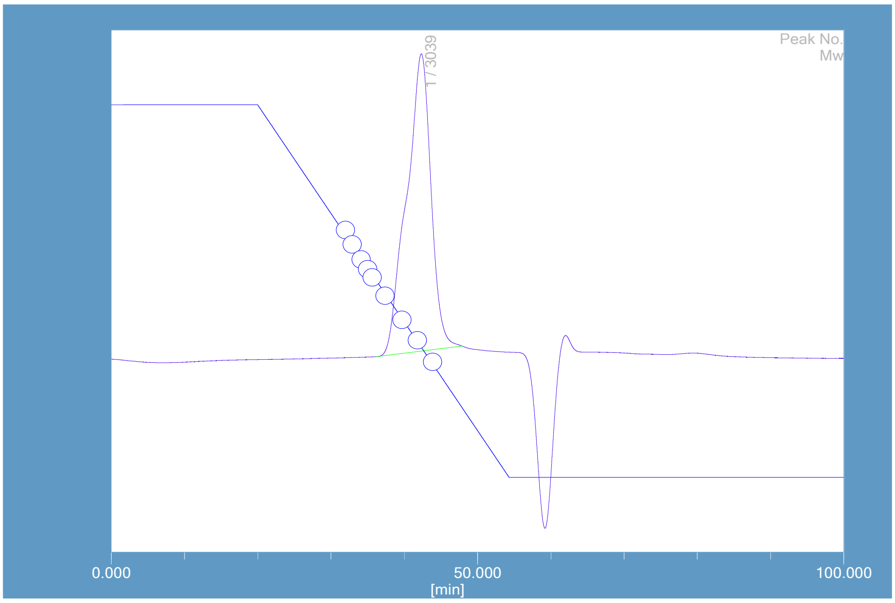

2022-07-01
: RSLT0002
: cg-01-34CG

: ChromatogramRI (-306.330 - 521.930 [mV])
: Calibration CurveRI (-1.800 - 10.800 [LogM])

-RI- MN: 1160 Mw: 3039 Mz: 7140 Mw/Mn: 2.619

Figure S6. GPC trace (RI detection, HFIP and 3.0 mg/mL CF_3COOK mobile phase) of poly(BocAz) synthesized from a polymerization performed with a **BocAz**:BuN(K)Ts ratio of 20:1.

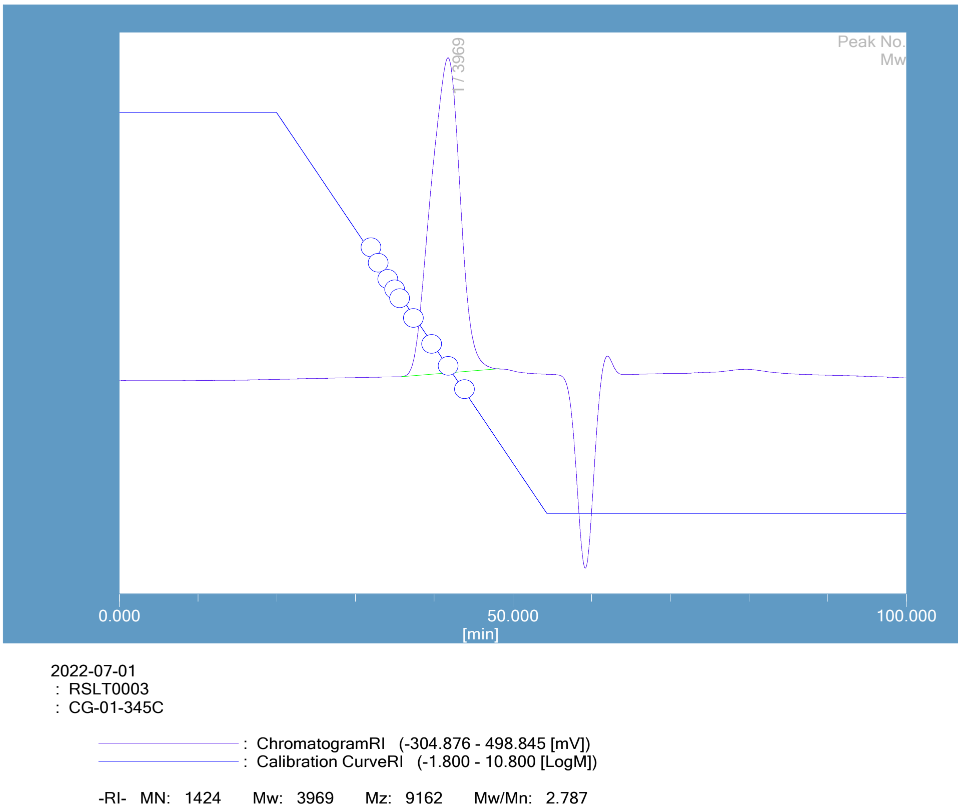

Figure S7. GPC trace (RI detection, HFIP and 3.0 mg/mL CF_3COOK mobile phase) of poly(BocAz) synthesized from a polymerization performed with a **BocAz**:BuN(K)Ts ratio of 40:1.

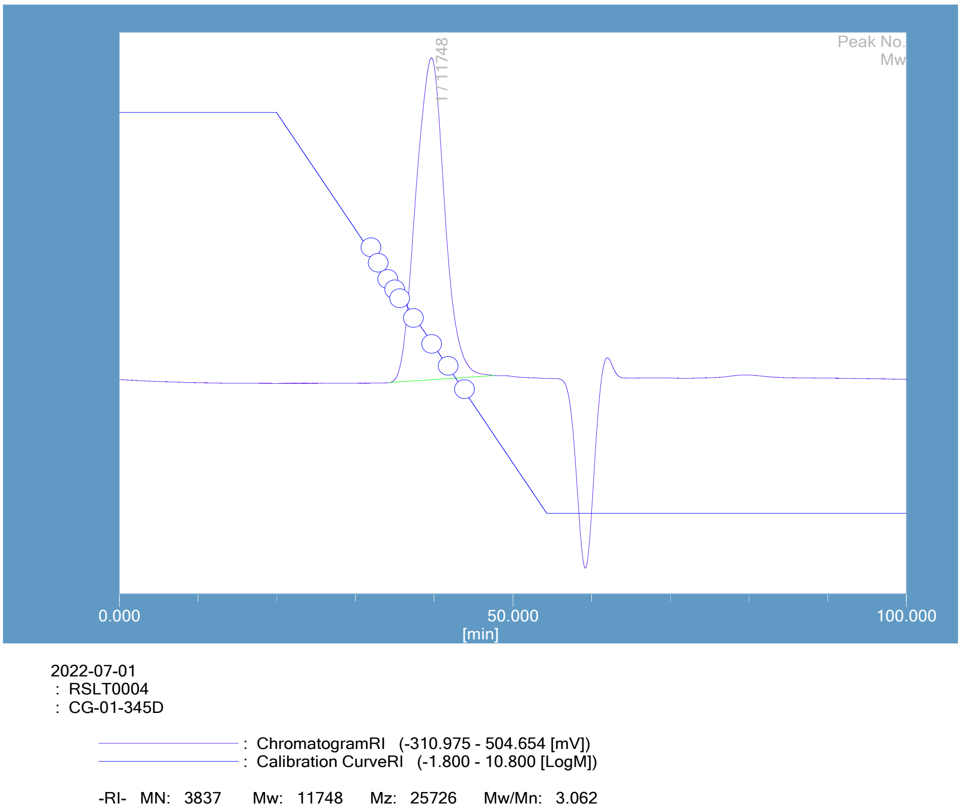

Figure S8. GPC trace (RI detection, HFIP and 3.0 mg/mL CF_3COOK mobile phase) of poly(BocAz) synthesized from a polymerization performed with a **BocAz**:BuN(K)Ts ratio of 80:1.

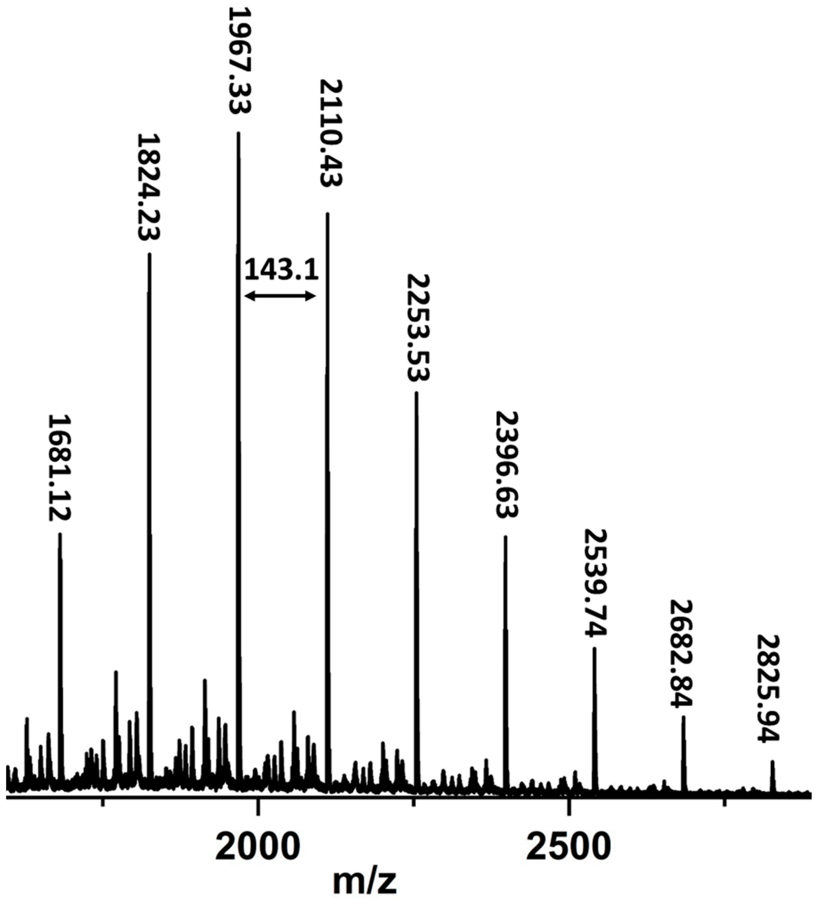

Figure S9. MALDI-ToF MS of the poly(BocAz). The signal at 2110 m/z matches a polymer chain with this formula $(C_{11}H_{16}NSO_2)(C_7H_{13}NO_2)_{13}H+Na^+$.

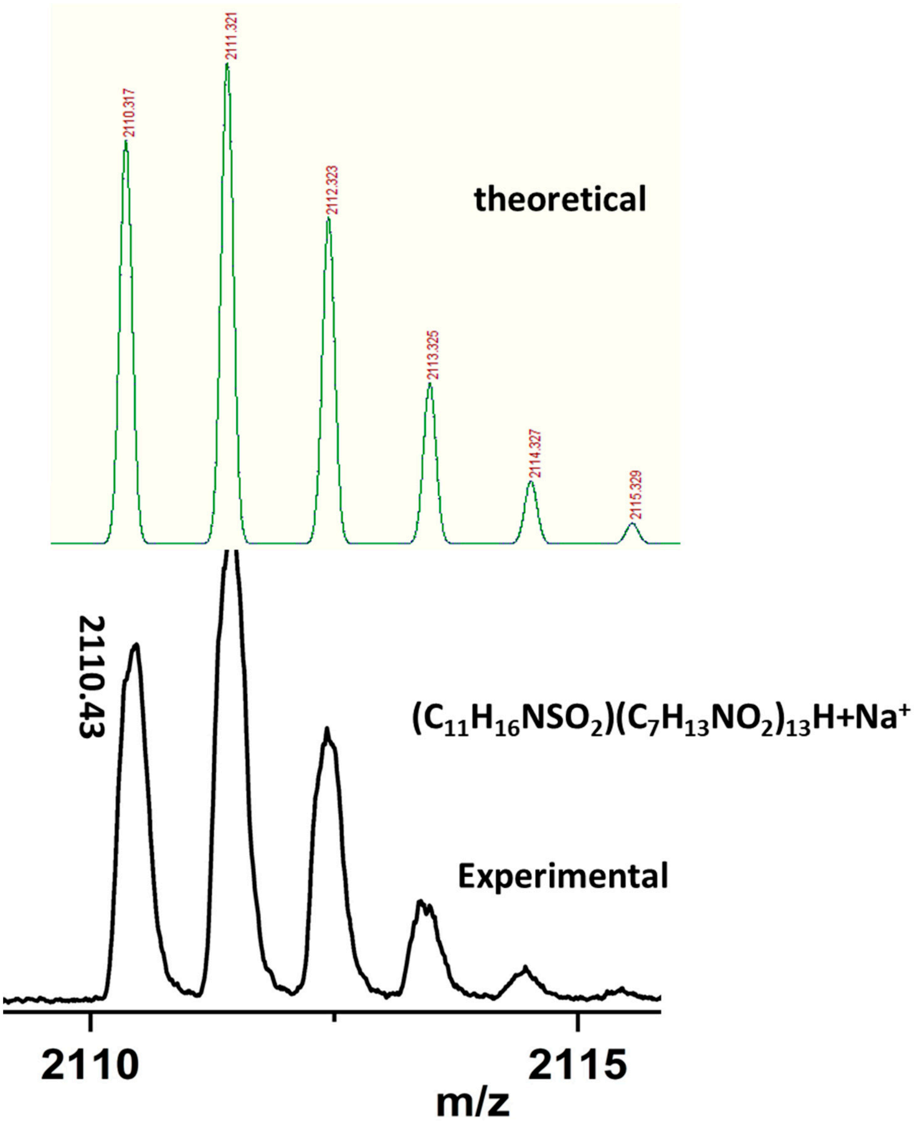

Figure S10. Comparison of the isotopic pattern from a MALDI-ToF MS of the poly(BocAz) (bottom) to theoretical(top). The signal at 2210 m/z matched a polymer chain with this formula: $(C_{11}H_{16}NSO_2)(C_7H_{13}NO_2)_{13}H+Na^+$.
